# DNA methylation-based biomarkers for early detection of non-small cell lung cancer: an update

**DOI:** 10.1186/1476-4598-7-81

**Published:** 2008-10-23

**Authors:** Paul P Anglim, Todd A Alonzo, Ite A Laird-Offringa

**Affiliations:** 1 Departments of Surgery and of Biochemistry and Molecular Biology, Keck School of Medicine, Norris Comprehensive Cancer Center, University of Southern California, Los Angeles, CA 90089-9176, USA; 2 Department of Preventive Medicine, University of Southern California, Arcadia, CA, 91006, USA

## Abstract

Lung cancer is the number one cancer killer in the United States. This disease is clinically divided into two sub-types, small cell lung cancer, (10–15% of lung cancer cases), and non-small cell lung cancer (NSCLC; 85–90% of cases). Early detection of NSCLC, which is the more common and less aggressive of the two sub-types, has the highest potential for saving lives. As yet, no routine screening method that enables early detection exists, and this is a key factor in the high mortality rate of this disease. Imaging and cytology-based screening strategies have been employed for early detection, and while some are sensitive, none have been demonstrated to reduce lung cancer mortality. However, mortality might be reduced by developing specific molecular markers that can complement imaging techniques. DNA methylation has emerged as a highly promising biomarker and is being actively studied in multiple cancers. The analysis of DNA methylation-based biomarkers is rapidly advancing, and a large number of potential biomarkers have been identified. Here we present a detailed review of the literature, focusing on DNA methylation-based markers developed using primary NSCLC tissue. Viable markers for clinical diagnosis must be detectable in 'remote media' such as blood, sputum, bronchoalveolar lavage, or even exhaled breath condensate. We discuss progress on their detection in such media and the sensitivity and specificity of the molecular marker panels identified to date. Lastly, we look to future advancements that will be made possible with the interrogation of the epigenome.

## Background

Worldwide lung cancer kills over one million people each year, and as the leading cause of cancer death in men and second leading cause in women, it is a major health problem [1]. This disease is largely smoking-associated. While in developed countries smoking rates are decreasing, the use of tobacco products is increasing in developing countries. In combination with a spike in the number of lung cancer cases in never smokers, this ensures that lung cancer will remain a major health problem [1]. Clinically, lung cancer is divided into two subtypes, small cell lung cancer (SCLC) and non-small cell lung cancer (NSCLC). SCLC is the more aggressive subtype, and accounts for 10–15% of all cases. The remaining 85–90% of cases are classified as NSCLC, which is further histologically subdivided into four categories; adenocarcinoma (AD), squamous cell carcinoma (SQ), large cell carcinoma (LC) and 'others', for example cancers of neuroendocrine origin.

In the United States lung cancer is the number one cancer killer in both men and women, accounting for over 160,000 deaths each year [2]. Interestingly, it is not the most commonly diagnosed cancer; breast and prostate cancer have a higher incidence. A reason for this disparity is that early detection methods exist for breast and prostate cancer, and these are widely used in the population. As a result, the five-year survival rate is 89 and 99% (respectively) for these cancers, as opposed to a very low 15% for lung cancer [2]. When early stage lung cancer *is *detected, the survival rate can increase dramatically. For example, one report on detection of early stage cancers using low dose spiral computed tomography (LDSCT) described a ten-year survival rate of 88% [3]. While there is concern that LDSCT leads to overdiagnosis (detection of indolent cancers that would normally not lead to death), it is undisputed that effective early detection of lesions that would otherwise progress to invasive cancer could reduce lung cancer mortality. In an effort to achieve early detection many imaging and cytology-based strategies have been employed, however none have yet been proven effective. Molecular markers would provide an alternative approach and among them, DNA methylation alterations show great promise. Here we present an update of the field of DNA methylation markers for early lung cancer detection.

### Early detection of lung cancer

Original early detection methods for lung cancer were focused on screening using chest X-ray and sputum cytology. Randomized controlled trials demonstrated no reduction in mortality using these techniques [4,5]. The question has been raised as to whether these trials had enough statistical power to determine a mortality benefit [5,6]. The Prostate, Lung, Colorectal and Ovarian cancer trial currently being conducted by the National Cancer Institute is a larger trial and may conclusively reveal whether chest X-ray screening can reduce mortality [5]. As discussed later, studies of molecular instead of cytological changes in sputum samples appear promising [7].

Following the apparent failure of chest X-ray and sputum cytology as effective screening techniques, attention was focused on a more sensitive imaging method – Low Dose Spiral Computed Tomography (LDSCT). Several trials of LDSCT as a screening tool in high-risk populations have been conducted [8-14]. It is clear that LDSCT is more sensitive than chest X-ray [11,12], as it can detect non-calcified nodules as small as 1 mm. Such high sensitivity comes with a price. The number of non-calcified nodules detected is far greater than the number of actual cancers. A Mayo Clinic study in 1999 reported that <2.0% of non-calcified nodules detected were actually cancer [15]. This presents two potential problems for LDSCT as an early detection method. Firstly, there is the potential for many false positive results, which would result in low specificity if LDSCT were applied as a lung cancer screening tool. The second problem is that in order to determine which nodules are actually cancer, patients will require follow up procedures (further scans, possibly biopsies or resections). These are costly, invasive, and can result in patient morbidity and mortality. Crestanello *et al*. report that 9 out of 54 patients underwent surgery for benign nodules [16]. A review of seven studies by Diederich and Wormanns reported that 4–55% of patients had invasive procedures for benign lesions [6].

An increase in survival in LDSCT-screened lung cancer patients has been reported; the IELCAP study reports an 88% 10-year survival [3]. Many argue that the increased survival rate seen is due to an overdiagnosis bias. Using the Yankelevitz criteria of overdiagnosis – a tumor volume doubling time (VDT) of > 400 days [17] – 27% of the detected cancers in a study by Lindell *et al*. would be considered overdiagnosed [8]. In a review by Jett of a Japanese study, 33% of the cancers detected have a VDT of >400 days [18], and hence would be considered overdiagnosed [15]. Using a predictive model, Bach and colleagues recently examined the combined results of LDSCT screening trials from three centers. They found an excess number of cases diagnosed at each screening point compared to the predicted number, without a decline in the number of advanced cancers being detected. This supports the notion of overdiagnosis in LDSCT screening [19]. The true measure of efficacy of an early detection method is a reduction in mortality. Whether LDSCT screening in high-risk populations decreases lung cancer mortality remains unknown. The answer to this question will hopefully be provided by one of several ongoing randomized controlled trials (for example the US-based National Lung Screening Trial, and in the Netherlands, the Dutch Lung Cancer Screening Trial). The conclusions from such trials will determine the fate of LDSCT as an early detection strategy.

Another imaging-based early detection approach is autofluoresence bronchoscopy (AFB). This distinguishes between tumor and non-tumor tissue based on the tumor-specific change in tissue autofluoresence. AFB has been shown to be effective at detecting preneoplastic lesions and lung cancers [20]. The drawbacks of the method are that it is invasive, it mainly detects centrally located cancers [21], and it is not highly specific [21,22].

Since imaging techniques have not yet proven effective as an early detection method, a sensitive and specific screening strategy remains to be found. To fill this void, research focus has shifted to molecular approaches. The goal is to identify molecular markers (generally DNA, RNA or protein) that reflect characteristics of lethal tumors, and that can be exploited for early detection of these lesions at the pre-invasive stage. To function as molecular markers in a screening test, these molecules must be detectible in remote media. If molecular markers that allow detection of cancer are identified, they will require complementary highly sensitive imaging methods such as LDSCT to locate the cancer. Identified molecular markers could be potentially targeted by agents to help specifically enhance tumor imaging [23].

### DNA methylation

One highly promising molecular biomarker is DNA methylation. This enzymatic addition of a methyl group at the 5-position of the cytosine in a CpG (cytosine-guanine) dinucleotide is a normal process within cells. In cancer, despite a global hypomethylation, one observes hypermethylation in regions of the genome described as CpG islands [24,25]. These islands are present in almost half of all genes and are frequently promoter-associated [26]. The common occurrence of DNA hypermethylation in all types of cancer makes it an ideal biomarker, one that has been extensively investigated. An advantage of DNA methylation over protein-based markers is that it is readily amplifiable and easily detectable using PCR-based approaches. In addition, contrary to cancer-specific mutations, which could occur anywhere in a gene, cancer-specific DNA hypermethylation occurs in defined regions, usually in or near the promoter of genes. Thus, it is easy to devise targeted probes to measure this molecular alteration. Conveniently, these probes can be readily combined into panels, which is important because no single molecular alteration involved in cancer can be expected to be present in every cancer case. Thus DNA methylation at a single gene would likely allow detection of a subset of cancers. Assembly of a complementary panel of DNA methylation probes would therefore increase sensitivity [27,28]. Finally, it has been demonstrated that methylated DNA can be isolated from 'remote media' making it well-suited for non-invasive detection [29,30].

### Overview of DNA methylation analysis in NSCLC

In this review, we focus on DNA methylation-based biomarkers for early detection of NSCLC. Because NSCLC is the less aggressive lung cancer subtype, and accounts for 85–90% of all cases, its early detection holds the most promise for saving lives. A plethora of studies describing DNA methylation in non-small-cell lung cancer exist. These studies are summarized in three tables, which, due to their size, are attached to this manuscript as Additional files 1, 2, 3. Each file lists the relevant loci in alphabetical order. Additional file 1 lists information from studies of less than 20 loci. Additional file 2 lists the results of DNA methylation studies of 20 or more loci, or genome wide approaches. Lastly, Additional file 3 discusses loci studied in remote media from cancer patients. The contents of these tables are discussed in more detail below.

Initial DNA methylation studies in NSCLC focused on single loci (or a small number of well known loci) that were selected because of their potential functional role in cancer. The goals of these studies were a) to see if methylation was involved in lung cancer pathogenesis, or b) to determine if methylation of a given gene could be correlated with clinical factors, and hence serve as a prognostic marker. This led to the characterization of the DNA methylation status of many loci in NSCLC (listed in Additional file 1) [31-103]. The information gathered in these studies could be of clinical use for early detection, chemo prevention, diagnosis, treatment or prognosis [104]. Further studies employed panels of 8–19 loci (including these previously reported loci) for DNA methylation profiling [105-116] (see Additional file 1). This profiling was aimed at characterizing methylation status of many loci in NSCLC, or in some cases, at identifying loci with the highest methylation frequency in tumors *versus *non-tumor tissues, that could potentially be used as DNA methylation-based biomarkers of the disease.

Several loci identified in both types of studies (e.g. APC, CADM1, CDH1, CDH13, CDKN2A/p14(ARF), CDKN2A/p16, DAPK, FHIT, GSTP1, MGMT, MLH1 and RASSF1A) are reported to be methylated multiple independent times in the literature (reviewed in Additional file 1), and there is general consistency in the observed methylation frequency for these loci. Any inconsistencies could have multiple explanations, for example: the use of different techniques to study the methylation status, differences in the population in each study, and a difference in the subtype composition of the NSCLC collection studied.

To further characterize DNA methylation in NSCLC and facilitate the discovery of new markers, more recent studies have employed approaches that analyze large numbers of loci at one time. In these studies, the goal has been to identify DNA methylation-based discriminators of tumor and normal tissues, and tumor subtypes. Some of these approaches were targeted; the loci analyzed were selected based on their relationship to cancer. Other approaches were not designed to interrogate DNA methylation at specific loci, instead they examined the genome in greater depth and identified potentially informative DNA methylation biomarkers based on comparative profiling between tumor and non-tumor cells/tissues. The most promising loci to emerge from these reports are reviewed in Additional file 2. One targeted approach is to use sodium bisulfite-treated DNA (in which unmethylated Cs have been converted to Us) for semi-quantitative real time PCR (MethyLight) to examine methylation levels of multiple loci. Three recent reports described an examination of 27 loci in NSCLC [117], 28 loci in AD [27], and 42 loci in SQ [28] using MethyLight. All three studies described a panel of loci with the ability to sensitively and specifically detect cancer. Using a MALDI-TOF based approach 47 loci were studied in tumor and non-tumor tissues from 96 patients. Six loci (CLEC3B (previously TNA), MGP, RASSF1, SDK2, SERPINB5 and XAGE1A (previously GAGED2)) with statistically significantly higher methylation in tumor samples compared with non-tumor samples were identified [118]. A targeted microarray was used to study the methylation status of 59 loci (245 CpGs) and a set of loci to discriminate SQ (ADPRH (formerly ARH1), GP1BB, RARB and TMEFF2) and AD (CDKN1C, MGMT, TMEFF2) from normal lung was identified [119]. In a similar system, the promoter regions of 288 cancer-related genes were examined. Twenty-eight potential biomarker loci were identified and 5 were further examined in lung cancer tissues, yielding two (PAX3 and PYCARD/ASC) that showed frequent hypermethylation [120]. Restriction landmark genomic scanning (RLGS) allows interrogation of up to 2000 promoter sequences. In a study of 1184 CpG islands Dai *et al*. discovered 11 genes that are differentially methylated in cancer, two of which are methylated in ≥ 50% of tumors (GNAL and PDX1) [121]. A newer high throughput approach is the Illumina GoldenGate platform, which examines 1505 CpG sites in 807 genes. Recently a panel of loci that detects adenocarcinoma was discovered, of which 8 were further examined by bisulfite genomic sequencing (ASCL2, CDH13, HOXA11, HOXA5, NPY, RUNX3, TERT and TP73) [122]. A study using a large methylation microarray analyzed the promoter regions of 8091 loci, identifying the frequently methylated CIDEB gene [123].

While these approaches can be used to determine the DNA methylation status of large numbers of genes, a non locus-targeted approach that allows unbiased interrogation of DNA methylation in the genome could examine far more loci. This could yield additional biomarkers, as well as new information about general DNA methylation patterns in lung cancer. Using an expression microarray one can identify genes induced in cell lines treated with a DNA methylation inhibitor. Such genes are potential DNA methylation targets. Using this approach, Shames *et al*. identified 132 tumor-specific methylation candidates, 45 of which were further investigated, revealing seven potential lung cancer markers (ALDH1A3, BNC1, CCNA1, CTSZ, LOX, MSX1 and NRCAM) three of which showed frequent tumor-specific hypermethylation compared to non-tumors [124]. Cortese *et al*. used a different approach, studying the DNA methylation of genes that are differentially expressed in fetal vs. adult lung. Four loci (FGFR3, LAPTM5, MDK, MEOX2) were identified as aberrantly methylated in lung cancer, one with high frequency [125].

Using a methylated CpG island recovery assay coupled with microarray analysis (MIRA-microarray), Rauch *et al*. enriched for CpG regions and then hybridized this to a CpG microarray containing 12,192 CpG islands, ≥ 60% of which map to the 5' end of known or putative genes. Multiple highly methylated loci were identified, of which the top 50 were reported [126]. In follow-up studies they identified several loci as markers for SQ lung cancer [127], including HOXA7 and HOXA9 [128]. It is of note that while the non-targeted approaches have the potential to rapidly identify many more biomarkers, the candidate biomarker loci must still be validated in primary tumors using traditional approaches.

In general, there is not a large overlap between the top loci identified in the targeted and non-targeted approaches. Several frequently methylated loci identified in early studies, for example CDKN2A/p16, CDH13, MGMT and RASSF1 remain viable markers when assessed in a larger context, providing support for their role in cancer development/progression [27,28,118,119,122]. Methylation of genes that are occupied by transcriptionally repressive polycomb group protein in embryonic stem cells, such as members of the HOX and PAX families, was detected by targeted as well as genomic approaches. This reinforces the notion that these genes may be prone to cancer-specific methylation [129]. Further investigation of this group of genes is warranted.

Modest overlap between the top loci from the non-targeted studies is seen. This might be expected as each of these approaches differ in their methods of experimentation, data analysis and ranking of loci as biomarkers. It also indicates that further markers remain to be identified and that development of the optimal panel will require additional studies. Ongoing genome-wide analyses using a multitude of approaches will help solve this issue, but it is important that these analyses be carried out on all histological subtypes of lung cancer. As previously discussed, NSCLC is comprised of four histological sub-groups. The two most common subtypes, adenocarcinoma and squamous cell lung cancer, are quite distinct in both physical location and molecular profile [118,119,130-133]. They show differential methylation profiles as reported by Field *et al*. and Brena *et al*. [79,119]. Indeed work in our lab supports the notion of different methylation patterns in SQ and AD [27,28]. The distinct nature of AD and SQ means an optimal lung cancer methylation panel will probably require markers for both subtypes. Markers for LC and other minor NSCLC groups, such as neuroendocrine cancers, remain to be developed.

### DNA methylation in remote media

While using primary tissue to study methylation status is useful to discover potential biomarkers, this material is not non-invasively accessible and is therefore not useful for screening an at-risk population. The ideal system for early diagnosis is material collected in a non-invasive/minimally invasive way that will contain methylated DNA. For this, one looks to remote patient media – blood, naturally produced or induced sputum, exhaled breath-condensate (EBC, non-invasive), and bronchoalveolar lavage (BAL, semi-invasive). Multiple studies show that DNA methylation of certain loci can be detected in blood, sputum and BAL (Additional file 3). A few show that genetic alterations can be detected in EBC, as discussed below, although no published studies of DNA methylation detection in this medium exist.

The ideal remote medium is blood – it can be applied to all patients, both those at minimal and high risk, and is minimally invasive to obtain. It is reported that cancer patients have a higher level of circulating DNA than non-cancer cases [134], and that genetic [135-137], and epigenetic [138] alterations can be detected in said DNA. It is postulated that this DNA is released due to necrotic cell death [139]. Over 25 loci have been reported to be methylated in plasma/serum of NSCLC patients [29,41,45,52,55,60,140-144] (reviewed in Additional file 3). Several studies examined methylation in primary tumor material and corresponding plasma/serum, and in these cases methylation in blood was only seen in patients in which the primary tumor also exhibited methylation [52,60,142]. Many of the most promising markers from Additional file 1 and 2 have not yet been investigated in blood.

There are, however, caveats to detection of DNA methylation in blood. It is questioned as to whether there is enough methylated DNA in the blood to efficiently detect tumors at an early enough stage for curative resection. While DNA quantity may be low, ongoing research on more sensitive detection methods may overcome this issue. Another potential problem is that blood as a remote medium is not organ-specific; loci that are methylated in lung cancer may be methylated as well in other cancers, for example TNFRSF10C and D [113] TCF21 [36], RUNX3 [89], APC [145], FBN2 [68]. Thus, methylation of these loci in blood could point to cancer in any one of several organs. The best markers for lung cancer would therefore be ones that show methylation only in lung cancer. Given the recent focus on more genome wide approaches to study methylation in many cancer types, a comparison of DNA methylation profiles across cancer sites should soon be possible. An alternative to this is to complement DNA methylation marker screening with sensitive imaging techniques to identify the cancer site. Another option is to examine remote media that are more lung-specific.

Sputum is produced by increased bronchial secretions, and is commonly found in smokers, hence it can be used to screen high-risk populations. (In former or non-smokers, it is much more difficult to obtain, though it can be induced.) The advantages of sputum as a screening tool include its non-invasive procurement, and the fact that it contains cells from the lungs and lower respiratory tract. However, the material in sputum is from the center of the lungs, and it may not be as useful for the detection of adenocarcinoma, which generally occurs at the periphery. DNA methylation, mutations, and microsatellite alterations have been detected in sputum, indicating it is a useful source of tumor material [7,29,146]. Reports of DNA methylation in sputum are summarized in Additional file 3[29,57,59,77,80,97,113,140,147,148]. It has been demonstrated that promoter methylation in sputum increases with cancer risk [29], increases as the time to lung cancer decreases [147], and in the case of CDKN2A/p16 and/or MGMT, can be found in sputum up to 3 years before diagnosis of squamous cell lung cancer [149]. A study by Liu *et al*. using 50 matched tumor, plasma and sputum samples showed that CDKN2A/p16 hypermethylation is detected in 84% of tumors, and 76% of sputum samples from the same patients, demonstrating that this remote medium is potentially effective in detecting lung cancer [55]. However, whether this detection is applicable to all NSCLC subtypes remains to be determined.

Exhaled breath provides a source of materials that can reflect the disease state of the lungs. Breath condensate, comprised mostly of water vapors, also contains lipids, proteins, DNA and oxidation products – the levels of which may differ between healthy and diseased subjects [150]. Several studies report the utility of EBC in detection of asthma, chronic obstructive pulmonary disease (COPD) and cystic fibrosis [150]. EBC has also been used for NSCLC detection. Carpagnano *et al*. reported detection of the mitogenic factor endothelin-1 (ET1-1) in EBC of lung cancer patients. In a small study they showed a statistically significant difference in ET-1 levels between healthy controls and NSCLC patients, and between stage I-III and stage IV patients [151]. They have shown similar results when looking at interleukin-6 [152]. While these studies are protein-based, they do demonstrate the promise of EBC for early detection of lung cancer. Thus far, there are no published reports of DNA methylation detection in EBC, although two studies reported collecting sufficient DNA quantities to perform PCR-based assays for microsatellite alterations and *p53 *mutations [153,154]. Of concern is the fact that the p53 mutations detected in EBC differ from those found in the primary tumor from the same patient [153,154]. This raises concern regarding the origin of DNA obtained from EBC (it may also come from cells in the esophagus, throat or mouth) and its utility as a remote medium.

Bronchoalveolar lavage (BAL) is another potential screening material for early detection of lung cancer. While obtaining lavage fluid is not as invasive as a biopsy, it requires bronchoscopy. However, bronchoscopy is routinely performed in suspected lung cancer cases and lavage fluid can be easily obtained during this procedure. An advantage of BAL is that it allows *localized *harvesting of lung-specific material, so that the fluid can be expected to contain lung cancer cells and/or DNA. Several investigations of DNA methylation in BAL have been conducted [30,39,99,100,155-160] (Additional file 3). Results vary between studies. De Fraipont showed low levels of DNA methylation in BAL from tumor-bearing patients, indicating that this would not be a good medium for marker detection [157]. In contrast, Topalogu used a panel of loci and detected 68% of their tumor cases by examining DNA methylation in the corresponding BAL from the same patients [39]. Kim *et al*. also reported a good correlation between methylation in tumors and BAL, ranging from 39–61% for the five loci they analyzed [30]. DNA methylation has also been detected in control BAL from non-neoplastic patients [30,159,160]. The detection of DNA methylation in cancer-free patients is cause for concern if presence/absence of DNA methylation is being used as a diagnostic measure of cancer. However, if a quantitative assay to determine DNA methylation levels is applied, then one can determine a cut-off value, above which a sample would be considered positive, as was done by Grote *et al*. [159] and Schmiemann *et al*. [160].

The analyses of DNA methylation markers in remote media are still in their early stages, and although many show low sensitivity, the inclusion of more of the recently identified promising markers (Additional file 2) in future studies would likely boost detection of cancer cases. Published data so far supports the continued analysis of these fluids in search of an early detection method that can, at the very least, complement imaging-based screening of at risk subjects.

### Selection of DNA methylation-based biomarkers for early detection of NSCLC

While a plethora of loci are reported to serve as potential DNA methylation-based biomarkers for NSCLC, the important question is: Which should be chosen for further evaluation, and eventually for screening of subjects? When performing a screening test there are four potential outcomes. The first two of these, true-positive results (TP, those who test positive and actually have cancer), and true-negative results (TN, those who test negative and do not have cancer), are the desired outcome of a screening test. However, false-negative results (FN, those who have cancer but do not test positive), and false-positive results (FP, those who do not have cancer but test positive), could do serious harm to the screening populations. False negative results have the ramification of delaying diagnosis of the disease, hence endangering patients' lives, while false positive results significantly affect patient quality of life [161]. Sensitivity, defined as TP/(TP+FN), and specificity, defined as TN/(TN+FP), measure the balance of these results in the population. These measures can serve as the selection criteria to determine which potential biomarkers are pursued further. An ideal DNA methylation-based biomarker would be highly sensitive and specific in all populations studied, regardless of age, gender, ethnicity, risk factors and tumor stage. However, given the differences between NSCLC subtypes and smoking and non-smoking associated NSCLC, markers that function accurately in a subset of the population could also be of use. The likelihood of identifying a single marker with 100% sensitivity and specificity is negligible.

The methylation frequency for many loci examined in early studies is quite low in primary tumors (Additional file 1, for example, DAPK 16–47%, *p16 *23–81%, CDH13 28–48%, and RASSF1A 15–54%). If the methylation frequency is low, sensitivity will suffer as the locus yields too few cases. Even for the more frequently methylated loci listed in Additional file 2, one DNA methylation marker cannot be expected to detect all cases of a particular cancer. The way to address this problem is to study the DNA methylation status of multiple loci (a panel) in a sample population. To ensure high sensitivity individual loci in the panel should be highly penetrant, i.e. have a high frequency in the population, and be complementary, i.e. detect different tumor cases.

While ensuring high sensitivity is important, given very sensitive imaging approaches like LDSCT, the more critical issue in lung cancer screening is high specificity. False-positive results precipitate not only patient anxiety, but also follow up procedures that are invasive, costly, and have associated morbidity and mortality. The incidence for lung cancer in the United States is 79.4/100,000 in men and 52.6/100,000 in women [162]. This shows that less than 0.1% of the population will get lung cancer. Hence, a population-based screening using any marker with a specificity of less that 99.9% will detect more false positive cases than true positive ones. Such a marker therefore cannot function as a screening marker in the population at large. However, in current smokers the risk of lung cancer is greatly increased (incidence of over 230 per 100,000 for both men and women [163]), and the specificity of a marker can be slightly lower when screening is targeted to this high-risk group.

Sensitivity and specificity have been reported for several locus panels when examining methylation in DNA isolated from primary tissue. The area under the curve (AUC) of a receiver operating characteristic (ROC) curve is a measure of the ability of a continuous marker to accurately classify tumor and non-tumor tissue. Such a curve is a plot of sensitivity *vs *1 minus specificity values associated with all dichotomous markers that can be formed by varying the value threshold used to designate a marker "positive". An AUC of 1 corresponds to a marker with perfect accuracy, while an AUC of 0.5 corresponds to an uninformative marker. Shivapurkar *et al*. studied the DNA methylation of 11 loci to distinguish between NSCLC and adjacent non-tumor lung tissue. Using a logistic regression with a binary outcome indicator of tumor and non-tumor lung tissue, and a marker panel as covariates, they demonstrated that a combination of HS3ST2 (3OST2), DAPK and TNFRSF10C (DcR1) gave an ROC curve with an AUC of 0.959 when comparing tumor and adjacent non-tumor lung tissue. This implies that this combination of markers could sensitively and specifically detect lung cancer [113]. Ehrich *et al*. studied the methylation of 47 loci and developed a panel of 6 that could distinguish cancer from adjacent normal tissue with >95% sensitivity and specificity [118]. Feng *et al*. developed a panel of 8 loci, of which the presence of methylation of one gene was found in 80% of NSCLC tissues [117]. In an effort to develop markers for specific NSCLC subtypes, Tsou *et al*. reported a panel of 4 loci with 94% sensitivity and 90% specificity for AD [27], while Anglim *et al*. reported a panel of 4 loci that with 96.5% sensitivity and 93.3% specificity for SQ lung cancer [28]. Both reports compare DNA methylation in tumor and adjacent non-tumor tissue from the same patients. On a larger scale, Bibikova *et al*. identified 55 loci that distinguished AD from adjacent non-tumor lung with 100% sensitivity and 92% specificity [122]. These are all encouraging results, implying that DNA methylation detection could serve as a viable early detection biomarker, but these loci must be further validated in larger, racially/ethnicially and gender balanced independent populations in order to ensure equal functionality for all patients. Also, primary tissue would not be the source material tested in screening for early detection, hence, promising loci must be interrogated for their potential to sensitively and specifically detect cancer in remote media.

There are multiple reports of DNA methylation in blood, but not all assess the sensitivity and specificity of the loci. In those that do, it appears that detection in blood is commonly not sensitive [140,143]. For example, sensitivity ranged from 7–27% for CDH13, CDKN2A/p16, DAPK, GATA5, MGMT, PAX5α, PAX5β and RASSF1A in serum, but is much higher in sputum for the same samples [140]. One way in which investigators have tried to increase sensitivity is by defining a patient positive if a minimum number of loci are methylated. For example, Fujiwara *et al*. also described a low sensitivity of 49.5% when looking at methylation of at least one of 5 loci in serum (CDKN2A/p16, DAPK, MGMT, RARB and RASSF1A) but specificity was 85% [143]. Recently a report examining the methylation of CDH13, CDKN2A/p16, FHIT, RARB, RASSF1A and ZMYND10 (BLU) in which methylation of any 2 loci in plasma was considered cancer positive showed 73% sensitivity and 82% specificity [45]. This reinforces the notion that a panel of complementary loci is necessary. In an interesting report, Bearzatto *et al*. showed that combining CDKN2A methylation with microsatellite alterations in plasma increased sensitivity to 62%, and using CDKN2A methylation combined with circulating DNA levels increased specificity to 80%, as opposed to examining CDKN2A methylation alone [164]. While neither of these is ideal as a clinical test, it is of note that the marker panels need not consist solely of DNA methylation-based markers.

Many studies indicate that sputum could be a promising remote medium for early detection. Shivapurkar *et al*. described a combination of 4 loci, APC, CDKN2A/p16, HS3ST2 (3OST2), and RASSF1A that serve as a good panel for early detection of NSCLC in sputum, with an AUC of 0.8 [113]. Similarly, Li *et al*. reported a combination of FHIT and HYAL2 with 76% sensitivity and 85% specificity [7]. Wang et al. described MLH1 methylation in sputum to have 60% sensitivity and 86% specificity [77], and Belinsky showed that concomitant methylation of three or more of a panel of 6 loci resulted in 64% sensitivity and specificity [147]. In contrast, Cirincione *et al*. reported that 3 loci, CDKN2A/p16, RARβ2 and RASSF1A are of limited use in early detection of lung cancer using sputum as a remote medium [59].

Detection of DNA methylation in bronchoalveolar lavage is also documented. Grote *et al*. published two reports, using either APC or RASSF1A alone for NSCLC detection. In both cases there is low sensitivity (30 and 34% respectively) but high specificity (98.5 and 100% respectively) [156,158]. Using just CDKN2A/p16, Xie *et al*. describe a higher sensitivity (64%) than any other reports on DNA methylation in BAL when examining a single locus and a modest specificity (75%) [165]. Grote *et al*. explored the use of marker combinations in two studies. In the first they used CDKN2A/p16 and RARB2 in combination and showed 69% sensitivity and 87% specificity in their population [159]. In another study they applied a marker panel (APC, CDKN2A/p16, and RASSF1) to detect cancer in 247 patients, and reported 53% sensitivity and, in cases without a previous history of cancer, >99% specificity [160]. It is probable that the inclusion of more highly penetrant markers in such panels would increase sensitivity. This again highlights the need for a panel of markers, and underlines the need to combine molecular markers with imaging techniques.

## Conclusion

Lung cancer is responsible for a million cancer deaths per year worldwide, and its detrimental effects will continue to increase. Research focused on biomarker-based early detection has the potential to reduce mortality rates. What will it take to obtain functional DNA methylation markers for early lung cancer detection?

Sullivan-Pepe outlined the five phases of biomarker discovery[166]. The first phase, clinical exploratory, consists of identification of promising markers. Much work on identification of DNA-methylation based markers has already been done, as described in Additional files 1 and 2, and a number of markers has been carried forward to phase two, the clinical detection of established disease (Additional file 3). However, with the advent of new techniques, a thorough evaluation of the epigenome of all types of cancer will soon be possible. The pool of potential DNA methylation markers for lung cancer has by no means been exhausted, and it is expected that additional high penetrance markers will be identified. It will be important to examine DNA methylation in each of the major lung cancer histological subtypes and ensure the functionality of identified markers in lung cancers from both genders and all races/ethnic groups. In addition, given the fact that half of all new lung cancer cases arise in ex-smokers or never smokers [167], and the observed molecular differences between lung cancer from smokers and non-smokers [168], it would be important to ensure representation of lung cancer from never smokers in these marker screens. Standardization of epigenomic assay techniques and data analysis would facilitate comparisons of DNA methylation profiles between cancer types, which may allow the identification of true lung-cancer specific hypermethylation. Ideally, only reproducibly hypermethylated high penetrance DNA methylation markers should be carried forward to the analysis of systematically collected remote media (because remote media are such a valuable resource). The most promising markers can then be tested in retrospective longitudinal studies (phase three), in which materials collected prior to disease onset are examined. Studies of DNA methylation in sputum and BAL collected prior to diagnosis already look promising (e.g. [149,160]), and results can improve further with the inclusion of new high sensitivity/specificity marker panels. If results are promising, prospective screening studies (phase four) should follow to determine the extent and properties of detected disease and measure the false referral rate. Lastly, case control studies should be done to measure any effect on lung cancer mortality.

If a strong DNA methylation marker panel were developed, the manner in which it would be applied would depend on its sensitivity and specificity. It is unlikely that DNA methylation markers, or any molecular markers for that matter, would be used on their own. Instead, we envision that they will be applied in concert with high-resolution imaging. In the near future, the prospect of genome-wide interrogation of DNA methylation in lung cancer is extremely exciting. The resulting information may provide not only new candidate markers for early detection, but also for monitoring response to therapy and recurrence. In addition, methylation information could be linked to pathobiology and clinical characteristics, potentially providing indicators for treatment and prognosis. Much work remains to be done, but using epigenomics while building on the experience and materials obtained from prior studies, we are well armed to make non-invasive testing for early lung cancer detection a reality.

## Competing interests

The authors declare that they have no competing interests.

## Authors' contributions

PPA was involved in drafting the manuscript and generation of tables. TAA was involved in reviewing and editing the manuscript. IALO mentored PPA, and revised manuscript drafts. All authors reviewed and commented on the manuscript during its drafting and approved the final version.

## Supplementary Material

Additional file 1 Alphabetical list of all loci reported to be methylated in NSCLC in studies on single loci/small panels of loci. This table lists loci that were described to be methylated in studies of single loci, or small panels of loci, and describes in what fraction of samples the locus was methylated, from which source material the DNA was extracted, whether a specific NSCLC subtype was investigated, and the bibliography number for the reference. Click here for file

Additional file 2 Alphabetical list of selected loci of interest from studies using targeted or genome-wide approaches to examine DNA methylation at more than 20 loci. This table lists the loci of interest that were identified using approaches that examine many loci, the method used to identify them, the details concerning how many loci were examined, the fraction of tissues found to be methylated (where applicable), and the bibliography number for the reference. Click here for file

Additional file 3 Alphabetical list of genes for which DNA methylation status has been examined in remote media. This table lists loci for which DNA methylation status has been examined in remote media, the fraction of samples methylated, the remote medium used, the detection method used, and the bibliography number for the reference. Click here for file
