# Supplementary material for: DNA methylation-based biomarkers for early detection of non-small cell lung cancer: an update
Source: Mol Cancer. 2008 Oct 23;7:81. doi: 10.1186/1476-4598-7-81 (PMC2585582; doi:10.1186/1476-4598-7-81)
Supplement: Additional file 1 — Alphabetical list of all loci reported to be methylated in NSCLC in studies on single loci/small panels of loci. This table lists loci that were described to be methylated in studies of single loci, or small panels of loci, and describes in what fraction of samples the locus was methylated, from which source material the DNA was extracted, whether a specific NSCLC subtype was investigated, and the bibliography number for the reference. [file 1476-4598-7-81-S1.doc]

**Additional file 1**: Alphabetical list of all loci reported to be methylated in NSCLC in studies on single loci/small panels of loci

| **HUGO^a^** | **Gene Name^b^** | **Fraction Methylated^c^** | **Percent Methylated^d^** | **Material^e^** | **Subtype^f^** | **Method^g^** | **Ref^h^** |
| --- | --- | --- | --- | --- | --- | --- | --- |
| ACAT2 | Acetyl-Coenzyme A acetyltransferase 2 | 21/175 | 14 | TU | AD | MSP | 37 |
| ADAMTS8 | ADAM metallopeptidase with thrombospondin type 1 motif, 8 | 29/50 | 58 | TU |  | MSP | 75 |
|  |  | 16/24 | 67 | TU | AD | MSP | 75 |
|  |  | 13/26 | 50 | TU | SQ | MSP | 75 |
| AGT | Angiotensinogen | 21/99 | 21 | TU |  | MSP | 109 |
| AKAP12 | A kinase (PRKA) anchor protein (gravin) 12 | 67/175 | 39 | TU | AD | MSP | 37 |
| APC | Adenomatosis polyposis coli | 8/25 | 32 | TU |  | MSP | 105 |
|  |  | 7/7 | 100 | TU | AD | ML | 107 |
|  |  | 16/31 | 52 | TU | AD | MSP | 106 |
|  |  | 65/90 | 72 | TU |  | MSP | 38 |
|  |  | 22/48 | 46 | TU |  | MSP | 35 |
|  |  | 28/75 | 37 | TU |  | MSP | 110 |
|  |  | 48/99 | 48 | TU |  | MSP | 109 |
|  |  | 17/146 | 12 | TU |  | MSP | 115 |
|  |  | 12/40 | 30 | TU |  | QMSP | 113 |
|  |  | 17/31 | 55 | TU |  | QMSP | 39 |
|  |  | 86/91 | 95 | TU |  | QMSP | 40 |
|  |  | 24/105 | 25 | TU |  | MSP | 114 |
|  |  | 95/99 | 96 | TU |  | QMSP | 41 |
|  |  | n/a | n/a | TU |  | QAMA | 111 |
|  |  | 19/28 | 68 | TU |  | 3D Microarray | 108 |
| ATM | Ataxia telangiectasia mutated | 49/105 | 47 | TU |  | MSP | 114 |
| BCL2 | B-cell CLL/lymphoma 2 | 28/120 | 23 | TU |  | MSP | 42 |
|  |  | 19/48 | 40 | TU | AD | MSP | 42 |
|  |  | 9/72 | 13 | TU | SQ | MSP | 42 |
| BMP3 | Bone morphogenetic protein 3B | 45/91 | 46 | TU |  | MSP | 31 |
| BRCA1 | Breast cancer 1, early onset | 29/98 | 30 | TU |  | MSP | 46 |
|  |  | 1/22 | 4 | TU |  | MSP | 47 |
|  |  | 5/28 | 18 | TU |  | 3D Microarray | 108 |
| BRCA2 | Breast cancer 2, early onset | 41/98 | 42 | TU |  | MSP | 46 |
| CADM1 | Cell adhesion molecule 1 | 11/14 | 78.5 | TU |  | BGS | 94 |
|  |  | 21/48 | 44 | TU |  | BGS | 95 |
|  |  | 45/103 | 44 | TU |  | BGS | 96 |
|  |  | 29/68 | 43 | TU | AD | BGS | 96 |
|  |  | 14/26 | 54 | TU | SQ | BGS | 96 |
|  |  | 1/2 | 50 | TU | ADSQ | BGS | 96 |
|  |  | 1/7 | 14 | TU | LCC | BGS | 96 |
|  |  | n/a | n/a | TU |  | QAMA | 111 |
| CALCA | Calcitonin/calcitonin-related polypeptide, alpha | 6/7 | 86 | TU | AD | ML | 107 |
|  |  | n/a | n/a | TU |  | QAMA | 111 |
|  |  | 18/28 | 64 | TU |  | 3D Microarray | 108 |
| CCND2 | Cyclin D2 | 19/48 | 40 | TU |  | MSP | 35 |
|  |  | 25/61 | 47 | CL |  | MSP | 35 |
| CD9 | CD9 molecule | 3/19 | 13 | TU |  | MSP | 48 |
| CD44 | CD44 molecule | 5/28 | 18 | TU |  | 3D  Microarray | 108 |
| CDH1 | Cadherin-1 (E-cadherin) | 130/224 | 58 | TU |  | MSP | 49 |
|  |  | 2/7 | 29 | TU | AD | ML | 107 |
|  |  | 7/31 | 22 | TU | AD | MSP | 106 |
|  |  | 22/75 | 29 | TU |  | MSP | 110 |
|  |  | 19/107 | 18 | TU |  | MSP | 112 |
|  |  | 86/146 | 59 | TU |  | MSP | 115 |
|  |  | 5/40 | 12 | TU |  | QMSP | 113 |
|  |  | 27/31 | 87 | TU |  | QMSP | 39 |
|  |  | 69/105 | 66 | TU |  | MSP | 114 |
|  |  | 30/88 | 34.1 | TU |  | MSP | 109 |
|  |  | 3/28 | 11 | TU |  | 3D Microarray | 108 |
|  |  | 63/95 | 66 | TU |  | MSP | 98 |
| CDH13 | Cadherin-13 (H-Cadherin) | 130/305 | 43 | TU |  | MSP | 50 |
|  |  | 18/42 | 43 | TU |  | MSP | 33 |
|  |  | 15/30 | 50 | CL |  | MSP | 33 |
|  |  | 9/20 | 45 | TU |  | MSP | 51 |
|  |  | 5/7 | 71 | CL |  | MSP | 51 |
|  |  | 40/150 | 27 | TU |  | MSP | 105 |
|  |  | 70/146 | 48 | TU |  | MSP | 115 |
|  |  | 11/40 | 28 | TU |  | QMSP | 113 |
|  |  | 40/61 | 66 | TU |  | QMSP | 52 |
|  |  | 21/63 | 34 | TU |  | MSP | 45 |
|  |  | 26/88 | 29.5 | TU |  | MSP | 109 |
|  |  | 15/28 | 54 | TU |  | 3D Microarray | 108 |
| CDKN2A/p14 | Cyclin-dependent kinase inhibitor 2A | 4/46 | 9 | TU |  | MSP | 53 |
|  |  | 6/20 | 30 | TU | Central SQ | MSP | 54 |
|  |  | 10/40 | 25 | TU | Peripheral SQ | MSP | 54 |
|  |  | 5/31 | 16 | TU | AD | MSP | 106 |
|  |  | 9/107 | 8 | TU |  | MSP | 112 |
|  |  | 4/62 | 6 | TU |  | MSP | 47 |
| CDKN2A/p16 | Cyclin-dependent kinase inhibitor 2A | 22/54 | 41 | TU |  | MSP | 53 |
|  |  | 64/122 | 52.5 | TU |  | MSP | 56 |
|  |  | 11/18 | 61.1 | TU | SQ | MSP | 57 |
|  |  | 4/13 | 30.7 | TU | SQ | MSP | 58 |
|  |  | 8/20 | 40 | TU | Central SQ | MSP | 54 |
|  |  | 15/40 | 48 | TU | Peripheral SQ | MSP | 54 |
|  |  | 49/224 | 21.9 | TU |  | MSP | 49 |
|  |  | 44/150 | 29 | TU |  | MSP | 105 |
|  |  | 6/7 | 86 | TU | AD | ML | 107 |
|  |  | 14/31 | 45 | TU | AD | MSP | 106 |
|  |  | 58/119 | 49 | TU |  | MSP | 86 |
|  |  | 15/90 | 17 | TU |  | MSP | 38 |
|  |  | 23/75 | 31 | TU |  | MSP | 110 |
|  |  | 27/107 | 25 | TU |  | MSP | 112 |
|  |  | 22/99 | 22 | TU |  | MSP | 109 |
|  |  | 41/146 | 28 | TU |  | MSP | 115 |
|  |  | 18/40 | 45 | TU |  | QMSP | 113 |
|  |  | 12/29 | 41.4 | TU |  | MSP | 59 |
|  |  | 7/31 | 23 | TU |  | QMSP | 39 |
|  |  | 8/17 | 47 | TU |  | COBRA | 116 |
|  |  | 5/9 | 56 | CL |  | COBRA | 116 |
|  |  | 28/89 | 31 | TU |  | MSP | 47 |
|  |  | 41/105 | 39 | TU |  | MSP | 114 |
|  |  | 89/111 | 80.2 | TU |  | Semi-nested MSP | 55 |
|  |  | 48/61 | 79 | TU |  | QMSP | 52 |
|  |  | 73/92 | 79.3 | TU |  | QMSP | 60 |
|  |  | 33/63 | 53 | TU |  | MSP | 45 |
|  |  | n/a | n/a | TU |  | QAMA | 111 |
|  |  | 10/28 | 38 | TU |  | 3D Microarray | 108 |
|  |  | 48/75 | 64 | TU |  | MSP | 99 |
|  |  | 9/20 | 45 | TU |  | MSP | 100 |
| CDKN2B/p15 | Cyclin-dependent kinase inhibitor 2B | 4/20 | 20 | TU | Central SQ | MSP | 54 |
|  |  | 4/40 | 10 | TU | Peripheral SQ | MSP | 54 |
|  |  | 2/28 | 7 | TU |  | 3D Microarray | 108 |
| CHFR | Checkpoint with forkhead and ring finger domains | 7/37 | 19 | TU |  | MSP | 61 |
| CHEK2 | CHK2 checkpoint homolog | 9/9 | 100 | TU |  | MSP | 62 |
|  |  | 3/3 | 100 | CL |  | MSP |  |
| CST6 | Cystatin E/M | 9/19 | 50 | TU |  | MSP | 48 |
| DAB2IP | DOC-2/DAB2 interactive protein |  |  |  |  |  |  |
|  | m2a promoter region | 19/47 | 40 | CL |  | MSP | 63 |
|  |  | 26/70 | 37 | TU |  | MSP | 63 |
|  | m2b promoter region | 16/47 | 34 | CL |  | MSP | 63 |
|  |  | 25/70 | 36 | TU |  | MSP | 63 |
| DAPK1 | Death associated protein kinase 1 | 40/122 | 32.8 | TU |  | MSP | 64 |
|  |  | 2/31 | 6 | TU | AD | MSP | 106 |
|  |  | 15/90 | 17 | TU |  | MSP | 38 |
|  |  | 10/23 | 43 | CL |  | BGS | 34 |
|  |  | 12/32 | 37.5 | TU |  | BGS | 34 |
|  |  | 8/20 | 40 | TU | AD | BGS | 34 |
|  |  | 4/12 | 33 | TU | SQ | BGS | 34 |
|  |  | 21/75 | 28 | TU |  | MSP | 110 |
|  |  | 20/107 | 19 | TU |  | MSP | 112 |
|  |  | 37/146 | 25 | TU |  | MSP | 115 |
|  |  | 17/40 | 43 | TU |  | QMSP | 113 |
|  |  | 10/64 | 16 | TU |  | MSP | 47 |
|  |  | 24/105 | 23 | TU |  | MSP | 114 |
|  |  | 14/28 | 50 | TU |  | 3D Microarray | 108 |
|  |  | 26/75 | 35 | TU |  | MSP | 99 |
|  |  | 5/20 | 25 | TU |  | MSP | 100 |
| DBC1 | Deleted in bladder cancer 1 | n/a | n/a | TU |  | QAMA | 111 |
| DKK3 | Dickkopf homolog 3 | 32/238 | 13 | TU |  | MSP | 105 |
| DLC1 | Deleted in liver cancer 1 | 11/18 | 61 | TU |  | COBRA | 116 |
|  |  | 2/11 | 18 | CL |  | COBRA | 116 |
| DUOX1 | Dual oxidase 1 | 11/39 | 28 | TU | AD | MSP | 65 |
| DUOX2 | Dual oxidase 2 | 15/39 | 38 | TU | AD | MSP | 65 |
| EFEMP1 | Epidermal growth factor-containing fibulin like extracellular matrix protein 1 | 12/32 | 37.5 | TU |  | MSP | 66 |
| EGFL7 | EGF-like-domain, multiple 7 | 14/14 | 100 | TU |  | COBRA | 116 |
|  |  | 5/11 | 56 | CL |  | COBRA | 116 |
| ENG | Endoglin | 11/16 | 69 | TU |  | COBRA | 116 |
|  |  | 5/7 | 71 | CL |  | COBRA | 116 |
| Ep-CAM* | Epithelial cell adhesion molecule | 18/51 | 35 | TU | AD | MSP | 67 |
| ESR1 | Estrogen receptor 1 | 3/7 | 43 | TU | AD | ML | 107 |
|  |  | n/a | n/a | TU |  | QAMA | 111 |
|  |  | 15/28 | 54 | TU |  | 3D Microarray | 108 |
| ESR2 | Estrogen receptor 2 | 4/7 | 57 | TU | AD | ML | 107 |
| FBN2 | Fibrillin 2 | 14/16 | 88 | CL |  | MSP | 68 |
|  |  | 62/126 | 49 | TU |  | MSP |  |
| FHIT | Fragile Histidine Triad | 34/99 | 34 | TU |  | MSP | 70 |
|  |  | 28/91 | 31 | TU |  | MSP | 69 |
|  |  | 68/254 | 27 | TU |  | MSP | 70 |
|  |  | 117/224 | 52.2 | TU |  | MSP | 49 |
|  |  | 40/107 | 37 | TU |  | MSP | 112 |
|  |  | 24/63 | 39 | TU |  | MSP | 45 |
| GATA4 | GATA binding protein 4 | 42/63 | 67 | TU |  | MSP | 71 |
| GATA5 | GATA binding protein 5 | 26/63 | 41 | TU |  | MSP | 71 |
| GSTP1 | Glutathione S-transferase pi | 6/31 | 19 | TU | AD | MSP | 106 |
|  |  | 7/90 | 8 | TU |  | MSP | 38 |
|  |  | 1/75 | 1 | TU |  | MSP | 110 |
|  |  | 7/107 | 7 | TU |  | MSP | 112 |
|  |  | 15/99 | 15 | TU |  | MSP | 109 |
|  |  | 3/146 | 2 | TU |  | MSP | 115 |
|  |  | 3/31 | 10 | TU |  | QMSP | 39 |
|  |  | 2/21 | 9 | TU |  | MSP | 47 |
|  |  | 2/7 | 29 | TU | AD | ML | 107 |
| HOXA7 | Homeobox A7 | 10/22 | 45 | TU | SQ | COBRA | 72 |
| HOXA9 | Homeobox A9 | 15/22 | 68 | TU | SQ | COBRA | 72 |
| HRASLS | HRAS-like suppressor | 19/61 | 31.1 | TU | AD | MSP | 72 |
| HS3ST2 | Heparan sulfate D-glucosaminyl 3-O-sulfotransferase | 28/40 | 70 | TU |  | QMSP | 113 |
| HT1RB | 5-hydroxytryptamine receptor 1B | 14/20 | 70 | TU | SQ | MS-RDA | 73 |
| IL20RA | Interleukin 20 receptor, alpha | 45/175 | 26 | TU | AD | MSP | 37 |
| LAMA3 | Laminin, alpha 3 | 12/20 | 60 | CL |  | MSP | 76 |
|  |  | 15/36 | 42 | TU |  | MSP | 76 |
|  |  | 11/19 | 58 | TU | AD | MSP | 76 |
|  |  | 4/15 | 27 | TU | SQ | MSP | 76 |
| LAMB3 | Laminin, beta 3 | 3/20 | 15 | CL |  | MSP | 76 |
|  |  | 9/36 | 25 | TU |  | MSP | 76 |
|  |  | 6/19 | 32 | TU | AD | MSP | 76 |
|  |  | 3/15 | 20 | TU | SQ | MSP | 76 |
| LAMC2 | Laminin, gamma 2 | 5/20 | 25 | CL |  | MSP | 76 |
|  |  | 8/36 | 22 | TU |  | MSP | 76 |
|  |  | 6/19 | 32 | TU | AD | MSP | 76 |
|  |  | 2/15 | 13 | TU | SQ | MSP | 76 |
|  |  | 54/146 | 37 | TU |  | MSP | 115 |
| MGMT | O^6^-methylguanine-DNA methyltransferase | 37/122 | 30.3 | TU |  | MSP | 56 |
|  |  | 7/7 | 100 | TU | AD | ML | 107 |
|  |  | 13/31 | 42 | TU | AD | MSP | 106 |
|  |  | 22/53 | 42 | TU | AD | MSP | 103 |
|  |  | 25/70 | 36 | TU | SQ | MSP | 103 |
|  |  | 22/107 | 21 | TU |  | MSP | 112 |
|  |  | 45/146 | 31 | TU |  | MSP | 115 |
|  |  | 12/31 | 39 | TU |  | QMSP | 39 |
|  |  | 18/83 | 21 | TU |  | MSP | 47 |
|  |  | 15/105 | 10 | TU |  | MSP | 114 |
|  |  | 34/90 | 38 | TU |  | OMSP | 102 |
|  |  | 11/75 | 15 | TU |  | MSP | 99 |
|  |  | 14/20 | 70 | TU |  | MSP | 100 |
| MINT1 | Methylated in Tumor 1 | 33/146 | 23 | TU |  | MSP | 115 |
| MINT31* | Methylated in Tumor 31 | 64/146 | 44 | TU |  | MSP | 115 |
| MINT32* | Methylated in Tumor 32 | 33/146 | 23 | TU |  | MSP | 115 |
| MLH1 | mutL homolog 1, colon cancer, nonpolyposis type 2 | 5/75 | 7 | TU |  | MSP | 110 |
|  |  | 18/99 | 18 | TU |  | MSP | 109 |
|  |  | 2/146 | 1 | TU |  | MSP | 115 |
|  |  | 62/105 | 59 | TU |  | MSP | 114 |
|  |  | 43/77 | 55.8 | TU |  | MSP | 77 |
|  |  | 8/28 | 29 | TU |  | 3D Microarray | 108 |
| MSH2 | mutS homolog 2, colon cancer, nonpolyposis type 1 | 18/99 | 18 | TU |  | MSP | 109 |
|  |  | 43/77 | 55.8 | TU |  | MSP | 77 |
| MT3 | Metallothionein 3 | 13/19 | 68 | TU |  | MSP | 48 |
| MTHFR | 5,10-methylenetetrahydrofolate reductase (NADPH) | 7/7 | 100 | TU | AD | ML | 107 |
| MYOD1 | Myogenic differentiation 1 | 7/7 | 100 | TU | AD | ML | 107 |
|  |  | 14/90 | 16 | TU |  | MSP | 38 |
|  |  | n/a | n/a | TU |  | QAMA | 111 |
| MYO18B | Myosin XVIIIB | 7/20 | 35 | TU |  | BGS | 78 |
|  |  | 8/47 | 17 | CL |  | BGS | 78 |
| NNAT | Neuronatin | 12/19 | 64 | TU |  | MSP | 48 |
| NRIP3 | Nuclear receptor interacting protein3 | 6/19 | 32 | TU |  | MSP | 48 |
| OLIG1 | Oligodentrocyte transcription factor 1 | 26/41 | 63 | TU |  | COBRA | 79 |
| OXTR | Oxytocin receptor | 1/19 | 6 | TU |  | MSP | 48 |
| PAX5α* | Paired box 5 alpha | 9/11 | 82 | CL |  | MSP | 80 |
|  |  | 33/48 | 68.8 | TU |  | MSP | 80 |
|  |  | 16/25 | 64 | TU | AD | MSP | 80 |
|  |  | 17/23 | 74 | TU | SQ | MSP | 80 |
| PAX5β* | Paired box 5 beta | 9/11 | 82 | CL |  | MSP | 80 |
|  |  | 27/48 | 56 | TU |  | MSP | 80 |
|  |  | 13/25 | 52 | TU | AD | MSP | 80 |
|  |  | 14/23 | 61 | TU | SQ | MSP | 80 |
| PER1 | Period homolog 1 | 2/6 | 33 | TU |  | BGS | 81 |
| PGF | Placenta growth factor | 22/22 | 100 | TU |  | MSP | 82 |
| PGR* | Progesterone receptor A | 7/7 | 100 | TU | AD | ML | 107 |
| PRKCDBP | Protein kinase C delta binding protein | 11/14 | 79 | TU |  | BGS | 93 |
|  |  | 44/107 | 41 | TU |  | MSP | 112 |
| PTGS2 | Prostaglandin-endoperoxide synthase 2 | 7/7 | 100 | TU | AD | ML | 107 |
|  |  | 11/20 | 55 | TU |  | MSP | 100 |
| PYCARD | PYD and CARD domain containing | 7/146 | 5 | TU |  | MSP | 115 |
| RAMP2 | Receptor activity modifying protein 2 | 14/32 | 43.7 | TU |  | MSP | 66 |
| RARB | Retinoic acid receptor, beta | 48/150 | 32 | TU |  | MSP | 105 |
|  |  | 43/107 | 40 | TU |  | MSP | 112 |
|  |  | 92/146 | 63 | TU |  | MSP | 115 |
|  |  | 34/63 | 54 | TU |  | MSP | 45 |
|  |  | 53/75 | 71 | TU |  | MSP | 99 |
|  |  | 8/20 | 40 | TU |  | MSP | 100 |
| RARβ2* | Retinoic acid receptor, beta 2 | 7/31 | 22 | TU | AD | MSP | 106 |
|  |  | 138/342 | 40 | TU |  | MSP | 70 |
|  |  | 3/31 | 10 | TU |  | QMSP | 39 |
|  |  | 19/29 | 65.5 | TU |  | MSP | 59 |
|  |  | n/a | n/a | TU |  | QAMA | 111 |
| RASSF1 | Ras association (RalGDS/AF-6) domain family 1 | 7/7 | 100 | TU | AD | ML | 107 |
|  |  | 5/31 | 16 | TU | AD | MSP | 106 |
|  |  | 71/146 | 49 | TU |  | MSP | 115 |
|  |  | 6/28 | 21 | TU |  | 3D Microarray | 108 |
|  |  | 47/116 | 40.5 | TU |  | MSP | 84 |
|  |  | 17/35 | 48.6 | TU | AD | MSP | 84 |
|  |  | 30/81 | 37 | TU | SQ | MSP | 84 |
|  |  | 46/119 | 39 | TU |  | MSP | 85 |
|  |  | 57/122 | 46.7 | TU |  | MSP | 64 |
|  |  | 8/25 | 32 | TU |  | MSP | 106 |
|  |  | 83/178 | 47 | TU |  | MSP | 32 |
|  |  | 44/138 | 32 | TU |  | MSP | 44 |
|  |  | 17/61 | 27.8 | TU | AD | MSP | 72 |
|  |  | 32/75 | 43 | TU |  | MSP | 110 |
|  |  | 40/99 | 40 | TU |  | MSP | 109 |
|  |  | 18/40 | 45 | TU |  | QMSP | 113 |
|  |  | 14/31 | 45 | TU |  | QMSP | 39 |
|  |  | 15/29 | 51.7 | TU |  | MSP | 59 |
|  |  | 7/16 | 44 | TU |  | COBRA | 116 |
|  |  | 10/14 | 71 | CL |  | COBRA | 116 |
|  |  | 34/107 | 32 | TU |  | MSP | 87 |
|  |  | 16/105 | 15 | TU |  | MSP | 114 |
|  |  | 30/63 | 48 | TU |  | MSP | 45 |
|  |  | n/a | n/a | TU |  | QAMA | 111 |
| RASSF2 | Ras association (RalGDS/AF-6) domain family 2 | 22/50 | 44 | TU |  | MSP | 83 |
|  |  | 33/106 | 31 | TU |  | MSP | 84 |
| RASSF5 | Ras association (RalGDS/AF-6) domain family 5 | 17/61 | 27.8 | TU | AD | MSP | 72 |
| RBP1 | Retinol binding protein 1, cellular | 19/150 | 13 | TU |  | MSP | 105 |
| RECK | reversion-inducing-cysteine-rich protein with Kazal motifs | 35/55 | 53.6 | TU |  | MSP | 88 |
| RPRM | Reprimo, TP53 dependent G2 arrest mediator candidate | 49/150 | 33 | TU |  | MSP | 105 |
| ROBO | Roundabout, axon guidance receptor, homology 1 | 15/32 | 46.8 | TU |  | MSP | 66 |
| RUNX3 | Runt-related transcription factor 3 | 6/25 | 24 | TU |  | MSP | 89 |
|  |  | 3/11 | 27 | TU | AD | MSP | 89 |
|  |  | 2/11 | 18 | TU | SQ | MSP | 89 |
|  |  | 1/11 | 9 | TU | LC | MSP | 89 |
|  |  | 15/75 | 20 | TU |  | MSP | 110 |
| SCGB3A1 | Secretoglobin, family 3A, member 1 | 95/339 | 28 | TU |  | MSP | 73 |
|  |  | 51/199 | 26 | TU | AD | MSP | 73 |
|  |  | 42/132 | 32 | TU | SQ | MSP | 73 |
|  |  | 2/8 | 25 | TU | LCC | MSP | 73 |
| SEMA3B | Sema domain, immunoglobulin domain (Ig), short basic domain, secreted, (semaphorin) 3B | 65/138 | 47 | TU |  | MSP | 44 |
| SEMA3G | Sema domain, immunoglobulin domain (Ig), short basic domain, secreted, (semaphorin) 3G | 13/14 | 93 | TU |  | COBRA | 116 |
|  |  | 6/11 | 55 | CL |  | COBRA | 116 |
| SFRP1 | Secreted Frizzled Related Protein 1 | 20/31 | 64 | TU | AD | MSP | 106 |
|  |  | 15/29 | 52 | CL |  | MSP | 92 |
|  |  | 44/80 | 55 | TU |  | MSP | 92 |
|  |  | 111/146 | 76 | TU |  | MSP | 115 |
|  |  | 81/238 | 34 | TU |  | MSP | 91 |
| SFRP2 | Secreted Frizzled Related Protein 2 | 123/146 | 84 | TU |  | MSP | 115 |
|  |  | 123/238 | 52 | TU |  | MSP | 91 |
| SFRP4 | Secreted Frizzled Related Protein 4 | 43/146 | 29 | TU |  | MSP | 115 |
| SFRP5 | Secreted Frizzled Related Protein 5 | 100/146 | 69 | TU |  | MSP | 115 |
|  |  | 78/238 | 33 | TU |  | MSP | 91 |
| SLIT2 | Slit homolog 2 | 16/16 | 100 | TU |  | COBRA | 116 |
|  |  | 3/3 | 100 | CL |  | COBRA | 116 |
| SLIT3 | Slit homolog 3 | 0/17 | 0 | TU |  | COBRA | 116 |
|  |  | 2/11 | 18 | CL |  | COBRA | 116 |
| SOCS1 | Suppressor of cytokine signaling 1 | 13/40 | 33 | TU |  | QMSP | 113 |
|  |  | 8/20 | 40 | TU |  | MSP | 105 |
| SOCS3 | Suppressor of cytokine signaling 2 | 7/8 | 87.5 | TU |  | MSP | 90 |
|  |  | 2/40 | 5 | TU |  | QMSP | 113 |
| SOX18 | SRY (sex determining region Y)-box 18 | 13/13 | 100 | TU |  | COBRA | 116 |
|  |  | 8/11 | 73 | CL |  | COBRA | 116 |
| SPARC | Secreted protein, acidic, cysteine-rich | 81/150 | 54 | TU |  | MSP | 106 |
| SYNE1 | Spectrin repeat containing, nuclear envelope 1 | 88/175 | 50 | TU | AD | MSP | 37 |
| TCF21 | Transcription factor 21 | 19/22 | 86 | TU |  | RLGS | 36 |
|  |  | 6/6 | 100 | TU |  | BGS | 36 |
|  |  | 142/175 | 81 | TU | AD | MSP | 37 |
|  |  | 30/40 | 75 | TU |  | QMSP | 113 |
| TIMP3 | TIMP metallopeptidase inhibitor 3 | 3/7 | 43 | TU | AD | ML | 107 |
|  |  | 28/107 | 26 | TU |  | MSP | 112 |
|  |  | 2/15 | 13 | TU |  | COBRA | 116 |
|  |  | 2/7 | 29 | CL |  | COBRA | 116 |
|  |  | 4/21 | 19 | TU |  | MSP | 47 |
|  |  | 12/28 | 43 | TU |  | 3D Microarray | 108 |
| TIMP4 | TIMP metallopeptidase inhibitor 4 | 17/18 | 98 | TU |  | COBRA | 116 |
|  |  | 9/14 | 63 | CL |  | COBRA | 116 |
| TMEFF2 | Transmembrane protein with EGF-like and two follistatin-like domains 2 | 56/150 | 37 | TU |  | MSP | 105 |
| TNFRSF10C | Tumor necrosis factor receptor superfamily, member 10c, decoy without an intracellular domain | 4/40 | 10 | TU |  | MSP | 101 |
|  |  | 3/26 | 12 | TU | AD | MSP | 101 |
|  |  | 1/14 | 7 | TU | SQ | MSP | 101 |
|  |  | 9/40 | 23 | TU |  | QMSP | 113 |
| TNFRSF10D | Tumor necrosis factor receptor superfamily, member 10d, decoy with truncated death domain | 11/40 | 28 | TU |  | QMSP | 113 |
| WIF1 | WNT inhibitory factor 1 | 11/31 | 35 | TU | AD | MSP | 106 |
|  |  | 66/238 | 28 | TU |  | MSP | 91 |
| XRCC5 | X-ray repair complementing defective repair in Chinese hamster cells 5 | 19/98 | 20 | TU |  | MSP | 46 |
| ZMYND10 | Zinc finger MYND-type containing 10 | 26/145 | 18.6 | TU |  | MSP | 39 |
|  |  | 68/160 | 42 | TU |  | MSP | 115 |
|  |  | 42/138 | 30 | TU |  | MSP | 44 |
|  |  | 19/63 | 31 | TU |  | MSP | 45 |

Alphabetical list of all genes reported to be methylated in studies using less than 20 genes. ^a^All gene symbols are HUGO. In cases where the HUGO symbol has changed, the HUGO symbol is used and the symbol at the time of publication is in parenthesis. * Denotes loci for which HUGO symbols cannot be found ^b^All gene names are from www.genecards.org. ^c^Fraction methylated refers to the number of tumors showing DNA methylation, and ^d^percent methylated is derived from this. ^e^Tissue type is either TU, tumor, or CL, cell line. ^f^Subtype refers to studies were a specific subtype of NSCLC was analyzed. AD is adenocarcinoma, SQ is squamous cell carcinoma. ^g^Method is the technique used to evaluate DNA methylation. BGS -Bisulfite genomic sequencing, COBRA - Combined bisulfite restriction analysis, ML - MethyLight, MSP - Methylation Sensitive PCR, MS-RDA - Methylation sensitive-representational difference analysis, QAMA - Quantitative analysis of methylated alleles, QMSP - Quantitative MSP, RLGS - Restriction landmark genome scanning. ^h^Ref is the citation listing number in the bibliography and equates to the citation number in the text.
