# Supplementary material for: DNA methylation-based biomarkers for early detection of non-small cell lung cancer: an update
Source: Mol Cancer. 2008 Oct 23;7:81. doi: 10.1186/1476-4598-7-81 (PMC2585582; doi:10.1186/1476-4598-7-81)
Supplement: Additional file 2 — Alphabetical list of selected loci of interest from studies using targeted or genome-wide approaches to examine DNA methylation at more than 20 loci. This table lists the loci of interest that were identified using approaches that examine many loci, the method used to identify them, the details concerning how many loci were examined, the fraction of tissues found to be methylated (where applicable), and the bibliography number for the reference. [file 1476-4598-7-81-S2.doc]

**Additional file 2**: Alphabetical list of selected loci of interest from studies using targeted or genome-wide approaches to examine DNA methylation at more than 20 loci

| **HUGO^a^** | **Gene Name^b^** | **Method^c^** | **Details^d^** | **Fraction**  **Methylated^e^** | **Ref^f^** |
| --- | --- | --- | --- | --- | --- |
| ADPRH | ADP-ribosylarginine hydrolase | Microarray | 245 CpG positions in 59 candidate genes in 26 SQ, 22 AD, 26 AdjNTL |  | 119 |
| ALDH1A3 | Aldehyde dehydrogenase 1 family, member A3 | MSP | 132 genes induced by 5-azadC, 31 methylated, top 8 analyzed in 20 T vs. 20 AdjNTL lung | 9/20 | 124 |
| ASCL2 | Achaete-scute complex homolog 2 | Illumina/BGS | Examined 1536 CpG sites in 371 genes, identified 55-gene panel that is 92% sensitive and 100% specific on 12 adenocarcinoma & 12 AdjNTL test set. 8 CpG sites validated using BGS |  | 122 |
| BARHL2 | BarH-like homeobox 2 | MIRA/Microarray | AdjNTL on partial genomic tiling arrays, detailed analysis of gene subset on 20 SQ T/AdjNTL | 17/20 | 127 |
| BMP3 | Bone morphogenetic protein 3B | RGLS | 1184 CpG islands in 16 NSCLC T vs. AdjNTL T vs. AdjNTL |  | 121 |
| BNC1 | Basonuclin 1 | MSP | 132 genes induced by 5-azadC, 31 methylated, top 8 analyzed in 20 T vs. 20 AdjNTL lung | 18/20 | 124 |
| BVES | Blood vessel epicardial substance | ML | 27 genes on 49 paired NSCLC T and AdjNTL |  | 117 |
| CCNA1 | Cyclin A1 | MSP | 132 genes induced by 5-azadC, 31 methylated, top 8 analyzed in 20 T vs. 20 AdjNTL lung | 18/20 | 124 |
| CD34 | CD34 molecule | RGLS | 1184 CpG islands in 16 NSCLC T vs. AdjNTL |  | 121 |
| CDH1 | Cadherin-1 (E-cadherin) | ML | 27 genes on 49 paired NSCLC T and AdjNTL |  | 117 |
| CDH13 | Cadherin-13 (H-Cadherin) | Illumina/BGS | Examined 1536 CpG sites in 371 genes, identified 55-gene panel that is 92% sensitive and 100% specific on 12 adenocarcinoma & 12 adjNTL test set. 8 CpG sites validated using BGS |  | 122 |
|  |  | ML | 27 genes on 49 paired NSCLC T and AdjNTL |  | 117 |
| CD8B | CD8 antigen, beta polypeptide b1 chain | RGLS | 1184 CpG islands in 16 NSCLC T vs. AdjNTL |  | 121 |
| CDKN1C | Cyclin-dependent kinase inhibitor 1C | Microarray | 245 CpG positions in 59 candidate genes in 26 SQ, 22 AD, 26 AdjNTL |  | 119 |
| CDKN2A/p16 | Cyclin-dependent kinase inhibitor 2A | ML | Out of over 100 loci, 28 chosen for evaluation, 7 show p<<0.0001 in 51 AD T vs. 38AdjNTL |  | 27 |
| CDX2 | Caudal type homeobox 2 | ML | Out of over 100 loci, 28 chosen for evaluation, 7 show p<<0.0001 in 51 AD T vs. 38AdjNTL |  | 27 |
| CIDEB | Cell death-inducing DFFA-like effector b | COBRA | 8091 CpGs examined in lung cancer cell lines, validation of two genes in 8AD, 8SQ, 5 SCLC |  | 123 |
| CLEC3B | C-type lectin domain family 3, member B  (TNA) | MALDI-TOF | 47 gene promoter regions in 96 T with AdjNTL |  | 118 |
| CTSZ | Cathepsin Z | MSP | 132 genes induced by 5-azadC, 31 methylated, top 8 analyzed in 20 T vs. 20 AdjNTL lung | 10/20 | 124 |
| CYP1B1 | Cytochrome P450, family 1, subfamily B, polypeptide 1 | RGLS | 1184 CpG islands in 16 NSCLC T vs. AdjNTL T vs. AdjNTL |  | 121 |
| DAPK1 | Death associated protein kinase | ML | 27 genes on 49 paired NSCLC T and AdjNTL |  | 117 |
| EVX2 | Even-skipped homeobox 2 | MIRA/Microarray | AdjNTL on partial genomic tiling arrays, detailed analysis of gene subset on 20 SQ T/AdjNTL | 16/20 | 127 |
| FABP3 | Fatty acid binding protein 3, intestinal | MSP | 288 genes in lung cancer cell lines using microarray. 5 genes in 22 T vs. AdjNTL |  | 120 |
| FGFR3 | Fibroblast growth factor receptor 3 | BGS | Studies a subset of 453 differentially expressed genes in 12 AD+3SQ vs. 5 normal adult lung samples |  | 125 |
| GDNF | Glial derived neurotrophic factor | ML | Examined 42 candidate loci from 304 prescreened markers. 8 show p<3E10-5 in SQ Tumor vs. AdjNTL, 45 cases |  | 28 |
| GNAL | Guanine nucleotide binding protein (G protein), alpha activating activity polypeptide, olfactory type | RGLS | 1184 CpG islands in 16 NSCLC T vs. AdjNTL |  | 121 |
| GP1BB | Glycoprotein Ib (platelet), beta polypeptide | Microarray | 245 CpG positions in 59 candidate genes in 26 SQ, 22 AD, 26 AdjNTL |  | 119 |
| HOXA1 | Homeobox A1 | ML | Out of over 100 loci, 28 chosen for evaluation, 7 show p<<0.0001 in 51 AD T vs. 38AdjNTL |  | 27 |
| HOXA5 | Homeobox A5 | Illumina/BGS | Examined 1536 CpG sites in 371 genes, identified 55-gene panel that is 92% sensitive and 100% specific on 12 adenocarcinoma & 12 AdjNTL test set. 8 CpG sites validated using BGS |  | 122 |
| HOXA7 | Homeobox A7 | MIRA/Microarray | Cell line DNA studied using MIRA and tiling arrays. Confirmatory analysis in 22 T/AdjNTL cases from stage 1NSCLC | 10/22 | 128 |
| HOXA9 | Homeobox A9 | MIRA/Microarray | Cell line DNA studied using MIRA and tiling arrays. Confirmatory analysis in 22 T/AdjNTL cases from stage 1NSCLC | 15/22 | 128 |
| HOXA11 | Homeobox A11 | Illumina/BGS | Examined 1536 CpG sites in 371 genes, identified 55-gene panel that is 92% sensitive and 100% specific on 12 adenocarcinoma & 12 AdjNTL test set. 8 CpG sites validated using BGS |  | 122 |
| IRF7 | Interferon regulatory factor 7 | MSP | 288 genes in lung cancer cell lines using microarray. 5 genes in 22 T vs. AdjNTL |  | 120 |
| IRX2 | Iroquois homeobox 2 | MIRA/Microarray | AdjNTL on partial genomic tiling arrays, detailed analysis of gene subset on 20 SQ T/AdjNTL | 19/20 | 127 |
| KCNH5 | Potassium voltage-gated channel, subfamily H (eag-related), member 5 | ML | 27 genes on 49 paired NSCLC T and AdjNTL |  | 117 |
| LAPTM5 | Lysosomal associated multispanning membrane protein 5 | BGS | Studies a subset of 453 differentially expressed genes in 12 AD+3SQ vs. 5 normal adult lung samples |  | 125 |
| LHX2 | LIM homeobox 2 | MIRA/Microarray/MSP | Purification of methylated DNA using methyl-binding domains followed by microarray hybridization. Confirmatory analysis in primary NSCLC tumors | 7/12 | 126 |
| LHX4 | LIM homeobox 4 | MIRA/Microarray/MSP | Purification of methylated DNA using methyl-binding domains followed by microarray hybridization. Confirmatory analysis in primary NSCLC tumors | 6/12 | 126 |
| LOX | Lysyl oxidase | MSP | 132 genes induced by 5-azadC, 31 methylated, top 8 analyzed in 20 T vs. 20 AdjNTL lung | 19/20 | 124 |
| MDK | Midkine (neurite growth-promoting factor 2) | BGS | Studies a subset of 453 differentially expressed genes in 12 AD+3SQ vs. 5 normal adult lung samples |  | 125 |
| MEIS1 | Meis homeobox 1 | MIRA/Microarray | AdjNTL on partial genomic tiling arrays, detailed analysis of gene subset on 20 SQ T/AdjNTL | 17/20 | 127 |
| MEOX2 | Mesenchyme homeobox 2 | BGS | Studies a subset of 453 differentially expressed genes in 12 AD+3SQ vs. 5 normal adult lung samples |  | 125 |
| MGP | matrix Gla protein | MALDI-TOF | 47 gene promoter regions in 96 T with AdjNTL |  | 118 |
| MGMT | O^6^-methylguanine-DNA methyltransferase | Microarray | 245 CpG positions in 59 candidate genes in 26 SQ, 22 AD, 26 AdjNTL |  | 119 |
|  |  | ML | 27 genes on 49 paired NSCLC T and AdjNTL |  | 117 |
| MLH3 | mutL homolog 3, colon cancer, nonpolyposis type 2 | COBRA | 8091 CpGs examined in lung cancer cell lines, validation of two genes in 8AD, 8SQ, 5 SCLC |  | 123 |
| MSX1 | Msh homeobox 1 | MSP | 132 genes induced by 5-azadC, 31 methylated, top 8 analyzed in 20 T vs. 20 AdjNTL lung | 11/20 | 124 |
| MSX2 | Msh homeobox 2 | MIRA/Microarray | AdjNTL on partial genomic tiling arrays, detailed analysis of gene subset on 20 SQ T/AdjNTL | 19/20 | 127 |
| MTHFR | 5,10-methylenetetrahydrofolate reductase (NADPH) | ML | Examined 42 candidate loci from 304 prescreened markers. 8 show p<3E10-5 in SQ Tumor vs. AdjNTL, 45 cases |  | 28 |
| NPY | Neuropeptide Y | Illumina/BGS | Examined 1536 CpG sites in 371 genes, identified 55-gene panel that is 92% sensitive and 100% specific on 12 adenocarcinoma & 12 AdjNTL test set. 8 CpG sites validated using BGS |  | 122 |
| NRCAM | Neuronal cell adhesion molecule | MSP | 132 genes induced by 5-azadC, 31 methylated, top 8 analyzed in 20 T vs. 20 AdjNTL lung | 18/20 | 124 |
| NR2E1 | Nuclear receptor subfamily 2, group E, member 1 | MIRA/Microarray | AdjNTL on partial genomic tiling arrays, detailed analysis of gene subset on 20 SQ T/AdjNTL | 20/20 | 127 |
| ONECUT2 | One cut homeobox 2 | MIRA/Microarray | AdjNTL on partial genomic tiling arrays, detailed analysis of gene subset on 20 SQ T/AdjNTL | 14/20 | 127 |
| OPCML | Opioid binding protein/cell adhesion molecule-like | ML | Out of over 100 loci, 28 chosen for evaluation, 7 show p<<0.0001 in 51 AD T vs. 38AdjNTL |  | 27 |
|  |  | ML | Examined 42 candidate loci from 304 prescreened markers. 8 show p<3E10-5 in SQ Tumor vs. AdjNTL, 45 cases |  | 28 |
| OSR1 | Odd-skipped related 1 | MIRA/Microarray | AdjNTL on partial genomic tiling arrays, detailed analysis of gene subset on 20 SQ T/AdjNTL | 20/20 | 127 |
| OTX1 | Orthodenticle homeobox 1 | MIRA/Microarray | AdjNTL on partial genomic tiling arrays, detailed analysis of gene subset on 20 SQ T/AdjNTL | 20/20 | 127 |
|  |  | RGLS | 1184 CpG islands in 16 NSCLC T vs. AdjNTL |  | 121 |
| PAX3 | Paired box 3 | MSP | 288 genes in lung cancer cell lines using microarray. 5 genes in 22 T vs. AdjNTL |  | 120 |
| PAX6 | Paired box 6 | MIRA/Microarray | AdjNTL on partial genomic tiling arrays, detailed analysis of gene subset on 20 SQ T/AdjNTL | 17/20 | 127 |
| PAX8 | Paired box 8 | ML | Examined 42 candidate loci from 304 prescreened markers. 8 show p<3E10-5 in SQ Tumor vs. AdjNTL, 45 cases |  | 28 |
| PDX1 | Pancreatic and duodenal homeobox 1 | RGLS | 1184 CpG islands in 16 NSCLC T vs. AdjNTL |  | 121 |
| PTPRN2 | Protein tyrosine phosphatase, receptor type, N polypeptide 2 | ML | Examined 42 candidate loci from 304 prescreened markers. 8 show p<3E10-5 in SQ Tumor vs. AdjNTL, 45 cases |  | 28 |
| PITX2 | Paired-like homeodomain 2 | ML | Examined 42 candidate loci from 304 prescreened markers. 8 show p<3E10-5 in SQ Tumor vs. AdjNTL, 45 cases |  | 28 |
| PYCARD | PY and CARD domain containing (ASC) | MSP | 288 genes in lung cancer cell lines using microarray. 5 genes in 22 T vs. AdjNTL |  | 120 |
| RARB | Retinoic acid receptor, beta | Microarray | 245 CpG positions in 59 candidate genes in 26 SQ, 22 AD, 26 AdjNTL |  | 119 |
|  |  | ML | 27 genes on 49 paired NSCLC T and AdjNTL |  | 117 |
| RASSF1 | Ras association (RalGDS/AF-6) domain family 1 | MALDI-TOF | 47 gene promoter regions in 96 T with AdjNTL |  | 118 |
|  |  | ML | 27 genes on 49 paired NSCLC T and AdjNTL |  | 117 |
| RIPK3 | Receptor-interacting serine-threonine kinase 3 | MSP | 288 genes in lung cancer cell lines using microarray. 5 genes in 22 T vs. AdjNTL |  | 120 |
| RUNX3 | Runt-related transcription factor 3 | Illumina/BGS | Examined 1536 CpG sites in 371 genes, identified 55-gene panel that is 92% sensitive and 100% specific on 12 adenocarcinoma & 12 AdjNTL test set. 8 CpG sites validated using BGS |  | 122 |
| SDK2 | Sidekick homolog 2 | MALDI-TOF | 47 gene promoter regions in 96 T with AdjNTL |  | 118 |
| SERPINB5 | Serpin peptidase inhibitor, clade B (ovalbumin), member 5 | MALDI-TOF | 47 gene promoter regions in 96 T with AdjNTL |  | 118 |
| SLC16A3 | Solute carrier family 16, member 3 | RGLS | 1184 CpG islands in 16 NSCLC T vs. AdjNTL |  | 121 |
| TAL1 | T-cell acute lymphocytic leukemia 1 | RGLS | 1184 CpG islands in 16 NSCLC T vs. AdjNTL |  | 121 |
| TBR1 | T-box, brain, 1 | RGLS | 1184 CpG islands in 16 NSCLC T vs. AdjNTL |  | 121 |
| TCF21 | Transcription factor 21 | ML | Examined 42 candidate loci from 304 prescreened markers. 8 show p<3E10-5 in SQ Tumor vs. AdjNTL, 45 cases |  | 28 |
| TERT | Telomerase reverse transcriptase | Illumina/BGS | Examined 1536 CpG sites in 371 genes, identified 55-gene panel that is 92% sensitive and 100% specific on 12 adenocarcinoma & 12 AdjNTL test set. 8 CpG sites validated using BGS |  | 122 |
| TFAP2α | Transcription factor AP-2 alpha | MIRA/Microarray | 4 SQ vs. AdjNTL on partial genomic tiling arrays, detailed analysis of gene subset on 20 SQ T/AdjNTL | 19/20 | 127 |
| TLX1 | T-cell leukemia homeobox 1 | RGLS | 1184 CpG islands in 16 NSCLC T vs. AdjNTL |  | 121 |
| TMEFF2 | Transmembrane protein with EGF-like and two follistatin-like domains 2 | Microarray | 245 CpG positions in 59 candidate genes in 26 SQ, 22 AD, 26 AdjNTL | 59 | 119 |
| TNFRSF25 | Tumor necrosis factor receptor superfamily, member 25 | ML | Examined 42 candidate loci from 304 prescreened markers. 8 show p<3E10-5 in SQ Tumor vs. AdjNTL, 45 cases |  | 28 |
| TP73 | Tumor protein p73 | Illumina/BGS | Examined 1536 CpG sites in 371 genes, identified 55-gene panel that is 92% sensitive and 100% specific on 12 adenocarcinoma & 12 AdjNTL test set. 8 CpG sites validated using BGS |  | 122 |
| XAGE1A | X antigen family, member 1 | MALDI-TOF | 47 gene promoter regions in 96 T with AdjNTL |  | 118 |
| ZNF577 | Zinc finger protein 577 | MIRA/Microarray | AdjNTL on partial genomic tiling arrays, detailed analysis of gene subset on 20 SQ T/AdjNTL | 18/20 | 127 |

Alphabetical list of selected loci of interest from studies using targeted or genome wide approaches to examine methylation at many loci. ^a^All gene symbols are HUGO. In cases where the HUGO symbol has changed, the HUGO symbol is used and the symbol at the time of publication is in parenthesis in the next column. ^b^All gene names are from www.genecards.org. ^c^Method is the technique used to evaluate DNA methylation. BGS - Bisulfite genomic sequencing, COBRA - Combined bisulfite restriction analysis, MALDI-TOF - Matrix assisted laser desorption ionization time of flight, MIRA - Methylated CpG-Island recovery assay, ML - MethyLight, MSP - Methylation Sensitive PCR, MS-RDA - Methylation sensitive-representational difference analysis, RLGS - Restriction landmark genome scanning. ^d^Details describes the number of loci examined, the source material used and numbers of tumors and the subtype of cancer. AD is adenocarcinoma, SQ is squamous cell carcinoma. NSCLC is non-small cell lung cancer. T = tumor, AdjNTL is adjacent non-tumor lung. ^e^Fraction methylated refers to the number of tumors showing DNA methylation. ^f^Ref is the citation listing number in the bibliography and equates to the citation number in the text.
