# Supplementary material for: DNA methylation-based biomarkers for early detection of non-small cell lung cancer: an update
Source: Mol Cancer. 2008 Oct 23;7:81. doi: 10.1186/1476-4598-7-81 (PMC2585582; doi:10.1186/1476-4598-7-81)
Supplement: Additional file 3 — Alphabetical list of genes for which DNA methylation status has been examined in remote media. This table lists loci for which DNA methylation status has been examined in remote media, the fraction of samples methylated, the remote medium used, the detection method used, and the bibliography number for the reference. [file 1476-4598-7-81-S3.doc]

**Additional file 3**: Alphabetical list of all genes for which DNA methylation status has been examined in remote media

| **HUGO^a^** | **Gene Name^b^** | **Fraction Methylated^c^** | **Samples^d^** | **Material^e^** | **Method^f^** | **Ref^g^** |
| --- | --- | --- | --- | --- | --- | --- |
| APC | Adenomatosis polyposis coli | 1/24 | Cases | BAL | MSP | 157 |
|  |  | 5/17 | Cases | BAL | QMSP | 39 |
|  |  | 3/10 | Controls | BAL | QMSP | 39 |
|  |  | 110/155 | Cases | BAL | QMSP | 156 |
|  |  | 28/67 | Controls | BAL | QMSP | 156 |
|  |  | 14/67 | Cases | BAL | QMSP | 160 |
|  |  | 1/102 | Controls | BAL | QMSP | 160 |
|  |  | 42/89 | Cases | Plasma/  Serum | QMSP | 41 |
|  |  | 0/50 | Controls | Plasma/  Serum | QMSP | 41 |
|  |  | 3/13 | Cases | Sputum | QMSP | 113 |
|  |  | 1/25 | Controls | Sputum |  | 113 |
| BHLHB5 | Basic helix-loop-helix domain containing, class B, 5 | 12/98 | Cases | Sputum | Nested MSP | 147 |
|  |  | 11/92 | Controls | Sputum | Nested MSP | 147 |
| CDH1 | Cadherin-1 (E-cadherin) | 13/27 | Cases | BAL | QMSP | 39 |
|  |  | 3/10 | Controls | BAL | QMSP | 39 |
| CDH13 | Cadherin-13 (H-Cadherin) | 11/85 | Cases | BAL | MSP | 30 |
|  |  | 4/127 | Controls | BAL | MSP | 30 |
|  |  | 21/63 | Cases | Plasma | MSP | 45 |
|  |  | 6/36 | Controls | Plasma | MSP | 45 |
|  |  | 14/61 | Cases | Serum | QMSP | 52 |
|  |  | 3/53 | Cases | Serum | MSP | 143 |
|  |  | 27/98 | Cases | Sputum | Nested MSP | 147 |
|  |  | 23/92 | Controls | Sputum | Nested MSP | 147 |
|  |  | 19/72 | Cases | Sputum | MSP | 143 |
| CDKN2A/p16 | Cyclin-dependent kinase inhibitor 2A | 12/19 | Cases | BAL | MSP | 155 |
|  |  | 4/24 | Cases | BAL | MSP | 157 |
|  |  | 1/7 | Cases | BAL | QMSP | 39 |
|  |  | 0/10 | Controls | BAL | QMSP | 39 |
|  |  | 14/85 | Cases | BAL | MSP | 30 |
|  |  | 8/127 | Controls | BAL | MSP | 30 |
|  |  | 17/50 | Cases | BAL | QMSP | 159 |
|  |  | 0/64 | Controls | BAL | QMSP | 159 |
|  |  | 4/20 | Cases | BAL | MSP | 100 |
|  |  | 14/68 | Cases | BAL | MSP | 99 |
|  |  | 9/67 | Cases | BAL | QMSP | 160 |
|  |  | 0/102 | Controls | BAL | QMSP | 160 |
|  |  | 103/136 | Cases | Plasma | Semi-nested MSP | 55 |
|  |  | 77/105 | Cases | Plasma | Nested MSP | 60 |
|  |  | 24/63 | Cases | Plasma | MSP | 45 |
|  |  | 3/36 | Controls | Plasma | MSP | 45 |
|  |  | 11/44 | LC survivors | Plasma | Nested MSP | 29 |
|  |  | 16/121 | Smokers | Plasma | Nested MSP | 29 |
|  |  | 7/74 | Never Smokers | Plasma | Nested MSP | 29 |
|  |  | 16/61 | Cases | Serum | QMSP | 52 |
|  |  | 14/100 | Cases | Serum | MSP | 143 |
|  |  | 3/9 | Cases | Serum | MSP | 142 |
|  |  | 12/35 | Cases | Serum | QMSP | 164 |
|  |  | 0/15 | Controls | Serum | QMSP | 164 |
|  |  | 15/72 | Cases | Serum | MSP | 140 |
|  |  | 19/53 | LC survivors | Sputum | Nested MSP | 29 |
|  |  | 30/118 | Smokers | Sputum | Nested MSP | 29 |
|  |  | 39/98 | Cases | Sputum | Nested MSP | 147 |
|  |  | 25/92 | Controls | Sputum | Nested MSP | 147 |
|  |  | 71/95 | Cases | Sputum | Semi-nested MSP | 55 |
|  |  | 3/13 | Cases | Sputum | QMSP | 113 |
|  |  | 2/25 | Controls | Sputum | QMSP | 113 |
|  |  | 1/29 | Cases | Sputum | MSP | 59 |
|  |  | 20/112 | Controls | Sputum | MSP | 59 |
|  |  | 11/11 | Cases | Sputum | Nested MSP | 149 |
|  |  | 18/123 | Controls | Sputum | Nested  MSP | 149 |
|  |  | 6/22 | Cases | Sputum | MSP | 148 |
|  |  | 29/72 | Cases | Sputum | MSP | 140 |
| DAPK | Death associated protein kinase | 3/24 | Cases | BAL | MSP | 157 |
|  |  | 14/68 | Cases | BAL | MSP | 99 |
|  |  | 3/20 | Cases | BAL | MSP | 100 |
|  |  | 7/72 | Cases | Serum | MSP | 138 |
|  |  | 10/100 | Cases | Serum | MSP | 143 |
|  |  | 4/5 | Cases | Serum | MSP | 142 |
|  |  | 25/53 | LC Survivors | Sputum | Nested MSP | 29 |
|  |  | 21/118 | Smokers | Sputum | Nested MSP | 29 |
|  |  | 42/98 | Cases | Sputum | Nested MSP | 147 |
|  |  | 30/92 | Controls | Sputum | Nested MSP | 147 |
|  |  | 22/72 | Cases | Sputum | MSP | 140 |
| FHIT | Fragile Histidine Triad | 7/24 | Cases | BAL | MSP | 157 |
|  |  | 19/85 | Cases | BAL | MSP | 30 |
|  |  | 36/127 | Controls | BAL | MSP | 30 |
|  |  | 20/63 | Cases | Plasma | MSP | 45 |
|  |  | 7/36 | Controls | Plasma | MSP | 45 |
| GATA4 | GATA binding protein 4 | 48/98 | Cases | Sputum | Nested MSP | 147 |
|  |  | 42/92 | Controls | Sputum | Nested MSP | 147 |
| GATA5 | GATA binding protein 5 | 10/45 | Cases | Serum | MSP | 140 |
|  |  | 34/98 | Cases | Sputum | Nested MSP | 147 |
|  |  | 26/92 | Controls | Sputum | Nested MSP | 147 |
|  |  | 31/72 | Cases | Sputum | MSP | 140 |
| GSTP1 | Glutathione S-transferase pi | 1/3 | Cases | BAL | QMSP | 39 |
|  |  | 0/10 | Controls | BAL | QMSP | 39 |
|  |  | 1/2 | Cases | Serum | MSP | 142 |
| HLHP* | Unknown | 42/98 | Cases | Sputum | Nested MSP | 147 |
|  |  | 36/92 | Controls | Sputum | Nested MSP | 147 |
| HOXA9 | Homeobox A9 | 14/22 | Cases | Sputum | MSP | 148 |
| HS3ST2 | Heparan sulfate D-glucosaminyl 3-O-sulfotransferase | 5/13 | Cases | Sputum | QMSP | 113 |
|  |  | 3/25 | Controls | Sputum | QMSP | 113 |
| IGFBP3 | Insulin-like growth factor binding protein 3 | 25/98 | Cases | Sputum | Nested MSP | 147 |
|  |  | 30/92 | Controls | Sputum | Nested MSP | 147 |
| LAMC2 | Laminin, gamma 2 | 72/98 | Cases | Sputum | Nested MSP | 147 |
|  |  | 70/92 | Controls | Sputum | Nested MSP | 147 |
| MAGE A1 | Melanoma antigen family A, 1 | 11/22 | Cases | Sputum | MSP | 148 |
| MAGE B2 | Melanoma antigen family B, 2 | 9/22 | Cases | Sputum | MSP | 148 |
| MGMT | O^6^-methylguanine-DNA methyltransferase | 3/24 | Cases | BAL | MSP | 157 |
|  |  | 7/12 | Cases | BAL | QMSP | 39 |
|  |  | 0/10 | Controls | BAL | QMSP | 39 |
|  |  | 11/20 | Cases | BAL | MSP | 100 |
|  |  | 6/68 | Cases | BAL | MSP | 99 |
|  |  | 5/44 | LC survivors | Plasma | Nested MSP | 29 |
|  |  | 15/121 | Smokers | Plasma | Nested MSP | 29 |
|  |  | 2/74 | Never Smokers | Plasma | Nested MSP | 29 |
|  |  | 17/100 | Cases | Serum | MSP | 143 |
|  |  | 4/6 | Cases | Serum | MSP | 142 |
|  |  | 4/72 | Cases | Serum | MSP | 140 |
|  |  | 19/53 | LC survivors | Sputum | Nested MSP | 29 |
|  |  | 17/118 | Smokers | Sputum | Nested MSP | 29 |
|  |  | 23/72 | Cases | Sputum | MSP | 140 |
|  |  | 23/98 | Cases | Sputum | Nested MSP | 147 |
|  |  | 22/92 | Controls | Sputum | Nested MSP | 147 |
|  |  | 7/11 | Cases | Sputum | Nested MSP | 149 |
|  |  | 31/123 | Controls | Sputum | Nested  MSP | 149 |
| MLH1 | mutL homolog 1, colon cancer, nonpolyposis type 2 | 9/21 | Cases | Sputum | MSP | 77 |
| PAX5α* | Paired box 5 alpha | 8/45 | Cases | Serum | MSP | 140 |
|  |  | 21/53 | LC Survivors | Sputum | Nested MSP | 29 |
|  |  | 14/118 | Smokers | Sputum Sputum | Nested MSP | 29 |
|  |  | 29/98 | Cases | Sputum | Nested MSP | 147 |
|  |  | 24/92 | Controls | Sputum | Nested MSP | 147 |
|  |  | 22/72 | Cases | Sputum | MSP | 140 |
| PAX5β* | Paired box 5 beta | 3/53 | Cases | Serum | MSP | 140 |
|  |  | 13/53 | LC Survivors | Sputum | Nested MSP | 29 |
|  |  | 11/118 | Smokers | Sputum | Nested MSP | 29 |
|  |  | 41/98 | Cases | Sputum | Nested MSP | 147 |
|  |  | 32/92 | Controls | Sputum | Nested MSP | 147 |
|  |  | 22/72 | Cases | Sputum | MSP | 140 |
| PTGS2 (COX2) | Prostaglandin-endoperoxide synthase 2 | 5/20 | Cases | BAL | MSP | 100 |
| RARB | Retinoic acid receptor, beta | 13/85 | Cases | BAL | MSP | 30 |
|  |  | 16/127 | Controls | BAL | MSP | 30 |
|  |  | 3/20 | Cases | BAL | MSP | 100 |
|  |  | 48/68 | Cases | BAL | MSP | 99 |
|  |  | 23/63 | Cases | Plasma | MSP | 45 |
|  |  | 6/36 | Controls | Plasma | MSP | 45 |
|  |  | 6/100 | Cases | Serum | MSP | 143 |
| RARβ2* | Retinoic acid receptor, beta 2 | 8/29 | Cases | Sputum | MSP | 59 |
|  |  | 58/112 | Controls | Sputum | MSP | 59 |
|  |  | 0/3 | Cases | BAL | QMSP | 39 |
|  |  | 0/10 | Controls | BAL | QMSP | 39 |
|  |  | 27/50 | Cases | BAL | QMSP | 159 |
|  |  | 8/64 | Controls | BAL | QMSP | 159 |
|  |  | 27/67 | Cases | BAL | QMSP | 160 |
|  |  | 21/102 | Controls | BAL | QMSP | 160 |
| RASSF1 (RASSF1A) | Ras-association domain family 1A gene | 31/111 | Cases | BAL | QMSP | 158 |
|  |  | 0/46 | Controls | BAL | OMSP | 158 |
|  |  | 4/14 | Cases | BAL | QMSP | 39 |
|  |  | 3/10 | Controls | BAL | QMSP | 39 |
|  |  | 15/85 | Cases | BAL | MSP | 30 |
|  |  | 5/127 | Controls | BAL | MSP | 30 |
|  |  | 5/20 | Cases | BAL | MSP | 100 |
|  |  | 20/67 | Cases | BAL | QMSP | 160 |
|  |  | 0/102 | Controls | BAL | QMSP | 160 |
|  |  | 16/44 | LC survivors | Plasma | Nested MSP | 29 |
|  |  | 2/121 | Smokers | Plasma | Nested MSP | 29 |
|  |  | 2/74 | Never Smokers | Plasma | Nested MSP | 29 |
|  |  | 24/63 | Cases | Plasma | MSP | 45 |
|  |  | 4/36 | Controls | Plasma | MSP | 45 |
|  |  | 10/12 | Cases | Serum | Meth-DOP-PCR | 141 |
|  |  | 11/100 | Cases | Serum | MSP | 143 |
|  |  | 7/72 | Cases | Serum | MSP | 140 |
|  |  | 27/80 | Cases | Serum | MSP | 144 |
|  |  | 0/50 | Controls | Serum | MSP | 144 |
|  |  | 13/53 | LC survivors | Sputum | Nested MSP | 29 |
|  |  | 8/118 | Smokers | Sputum | Nested MSP | 29 |
|  |  | 12/98 | Cases | Sputum | Nested MSP | 147 |
|  |  | 6/92 | Controls | Sputum | Nested MSP | 147 |
|  |  | 5/13 | Cases | Sputum | QMSP | 111 |
|  |  | 2/25 | Controls | Sputum | QMSP | 111 |
|  |  | 1/29 | Cases | Sputum | MSP | 59 |
|  |  | 1/112 | Controls | Sputum | MSP | 59 |
|  |  | 19/72 | Cases | Sputum | MSP | 140 |
| SEMA3B | Sema domain, immunoglobulin domain (Ig), short basic domain, secreted, (semaphorin) 3B | 45/50 | Cases | BAL | QMSP | 159 |
|  |  | 23/25 | Controls | BAL | QMSP | 97 |
| SFRP1 | Secreted Frizzled Related Protein 1 | 68/98 | Cases | Sputum | Nested MSP | 147 |
|  |  | 71/92 | Controls | Sputum | Nested MSP | 147 |
| SOCS1 | Suppressor of cytokine signaling 1 | 6/20 | Cases | BAL | MSP | 100 |
| TCF21 |  | 7/13 | Cases | Sputum | QMSP | 97 |
|  |  | 0/25 | Controls | Sputum | QMSP | 97 |
| ZMYND10 | Zinc finger MYND-type containing 10 | 19/63 | Cases | Plasma | MSP | 45 |
|  |  | 5/36 | Controls | Plasma | MSP | 45 |

Alphabetical list of all genes whose DNA methylation status has been examined in remote media. ^a^All gene symbols are HUGO. In cases where the HUGO symbol has changed, the HUGO symbol is used and the symbol at the time of publication is in parenthesis. * Denotes loci for which HUGO symbols cannot be found ^b^All gene names are from www.genecards.org. ^c^Fraction methylated refers to the number of tumors showing DNA methylation. ^d^Samples is either Cases i.e. have tumor, or Controls, i.e. no tumor. ^e^Media refers to which remote media was used in this study, BAL is bronchoalveolar lavage. ^f^Method is the technique used to evaluate DNA methylation. MSP - Methylation Sensitive PCR, QMSP - Quantitative MSP. ^g^Ref is the citation listing number in the bibliography and equates to the citation number in the text.
